# Antibiotic pressure does not uncover intra-host heterogeneity of *Pseudomonas aeruginosa* in patients with chronic lung disease

**DOI:** 10.1186/s12866-026-05147-9

**Published:** 2026-05-21

**Authors:** Lisa Göpel, Espen E Groth, Sina Minke, Hinrich Schulenburg, Barbara Kalsdorf, Laura Kirchhoff, Leif Tueffers, Klaus F Rabe, Sébastien Boutin, Dennis Nurjadi, Jan Rupp

**Affiliations:** 1 Institute of Medical Microbiology, University of Luebeck and University Hospital Schleswig-Holstein Campus Lübeck, Lübeck, Germany; 2 Department of Pneumology, LungenClinic Grosshansdorf, Großhansdorf, Germany; 3 Airway Research Center North (ARCN), member of the German Center for Lung Research (DZL), Großhansdorf, Germany; 4 Department of Evolutionary Ecology and Genetics, Zoological Institute, Kiel University, Kiel, Germany; 5 Max Planck Institute for Evolutionary Biology, Plön, Germany; 6 Division of Clinical Infectious Diseases, Research Center Borstel, Leibniz Lung Center, Borstel, Germany; 7 German Center for Infection Research (DZIF), Partner Site Hamburg-Lübeck-Borstel-Riems, Borstel, Lübeck, Germany; 8 Medical Faculty, Kiel University, Kiel, Germany; 9 Infectious Disease Clinic, University of Luebeck and University Hospital Schleswig-Holstein, Lübeck, Germany

**Keywords:** *Pseudomonas aeruginosa*, Chronic obstructive pulmonary disease, Non-cystic fibrosis bronchiectasis, Antimicrobial resistance, Eradication therapy, Intra-host heterogeneity

## Abstract

*Pseudomonas aeruginosa* colonization and infection in patients with chronic obstructive pulmonary disease (COPD) or non-cystic fibrosis bronchiectasis (NCFBE) are associated with increased airway inflammation and mortality. *Pseudomonas*-active antibiotic therapy is a frequent therapeutic intervention in acute bacteria-driven exacerbation or when eradication of *P. aeruginosa* colonization is intended. However, the impact of intra-patient heterogeneity of *P. aeruginosa* on eradication outcomes remains unclear.

We enrolled 30 patients receiving inpatient care for COPD and/or NCFBE exacerbation with known or newly detected *P. aeruginosa* colonization into an observational study. The analysis cohort consisted of 22 participants receiving in-hospital antibiotic eradication therapy, from whom a total of 91 lower respiratory tract samples were collected prior to, during, and after antibiotic therapy. To explore intra-host clonal heterogeneity, a maximum of 10 randomly selected *P. aeruginosa* isolates were collected from all patient samples containing *Pseudomonas.*

*P. aeruginosa*-positive cultures were detected in 11 of 22 patients at baseline or during antibiotic therapy. Among these patients, short-term microbiological eradication, defined as a *Pseudomonas*-negative culture during or at the conclusion of antibiotic treatment, was achieved in 10 of 11 cases (90.9%). Extended antimicrobial susceptibility testing was performed on all isolates and indicated low levels of resistance in isolates obtained from patients before the initiation of eradication therapy and overall limited intra-host phenotypic resistance variability. Whole-genome sequencing of isolates from four patients with at least three longitudinal *P. aeruginosa*-positive samples confirmed colonization by a single clonal lineage in each case.

While concordance between phenotypic resistance and detected genetic resistance determinants was limited, overall resistance levels were low. Our data suggest that *P. aeruginosa* colonization in our cohort of COPD/NCFBE patients was predominantly associated with a single clonal lineage and with limited intra-host resistance heterogeneity. These findings, based on a small sample size, suggest that susceptibility testing of a single isolate could inform eradication therapy in selected patients. The high rate of short-term eradication success is consistent with the effectiveness of current antibiotic regimens. Yet, more comprehensive longitudinal studies with larger patient numbers and more diverse patient backgrounds are warranted to clarify persistence and re-infection dynamics.

**Trial registration**

German Clinical Trials Register (DRKS), DRKS00023975. Registered 28 December 2020. Retrospectively registered.

**Supplementary Information:**

The online version contains supplementary material available at 10.1186/s12866-026-05147-9.

## Introduction

*Pseudomonas aeruginosa* is an opportunistic pathogen causing acute or chronic infections in immunocompromised patients, including those with sepsis, burn wounds, diabetes, cystic fibrosis, chronic obstructive pulmonary disease (COPD), and non-cystic fibrosis bronchiectasis (NCFBE) [1, 2]. In patients with NCFBE or COPD, *P. aeruginosa* infections are associated with lung function decline and increased mortality [3, 4]. A multiregional epidemiological study on 22,053 COPD outpatients showed sputum isolation of *P. aeruginosa* in 4.1% of cases, and hospitalization for exacerbation occurred more frequently in *P. aeruginosa*-positive patients [5]. Another study reported a 27.1% (95/350) rate of *P. aeruginosa*-positive sputum samples collected from patients with NCFBE, also reporting higher risks of exacerbation requiring hospitalization in this group [6].

The management of *P. aeruginosa* airway colonization frequently involves the administration of antibiotic eradication therapy, which includes antipseudomonal antibiotics such as ciprofloxacin or piperacillin/tazobactam [7]. However, the effectiveness of eradication therapy is reduced in the presence of resistance mutations, the horizontal acquisition of resistance genes, and/or phenotypic resistance development through the formation of biofilms [8]. A recent study reported the presence of mixed strain populations of *P. aeruginosa* in lower respiratory tract samples of intensive care unit (ICU) patients. The administration of antibiotic therapy (ABT) resulted in an accelerated adaptation to antibiotic treatment in these patients due to the selection of pre-existing resistant strains, compared to the sporadic evolution of resistance observed in patients colonized by single strains [9]. Eklöf et al. studied the presence of *P. aeruginosa* in 23 patients with COPD (83%, 19/23) over one year of longitudinal sampling. For 18 out of 19 patients with recurrent *Pseudomonas* detection, the same clonal lineage was identified [10]. Although whole-genome analysis revealed genetic adaptation over time through mutations in genes associated with antibiotic resistance, no antimicrobial susceptibility testing for the isolates was performed. While these studies report on the intra-patient heterogeneity of *P. aeruginosa*, the effect on eradication outcomes remains insufficiently explored.

To further investigate the dynamics of *P. aeruginosa* isolates in COPD and NCFBE patients, we conducted an observational clinical study. Patients diagnosed with COPD and/or NCFBE receiving inpatient treatment for exacerbation with evidence of airway colonization with *P. aeruginosa* were enrolled at two respiratory centers in Northern Germany. Longitudinal sampling of the lower airways (sputum and/or bronchial aspirates), before and/or during antibiotic eradication therapy, if administered, was performed. Up to 10 randomly selected *Pseudomonas* isolates were collected from each sample containing *Pseudomonas*. This study allowed us to investigate changes in antimicrobial resistance of *P. aeruginosa* isolates during antibiotic eradication therapy, and to explore the intra-host heterogeneity of COPD/NCFBE patients colonized with *P. aeruginosa*.

## Methods

### Clinical study and sample collection

Between November 2020 and October 2023, patients receiving inpatient care for COPD and/or NCFBE exacerbation with known or newly detected *P. aeruginosa* colonization were enrolled into an observational study at two North-German respiratory centers (LungenClinic Grosshansdorf and Medical Clinic of the Research Center Borstel). The study was approved by the ethics committee of the University of Luebeck (AZ 20–295) and registered in the German Clinical Trials Register (DRKS) (Identifier: DRKS00023975).

Inclusion criteria were: Age 18 or older, diagnosis of COPD and/or NCFBE, proven airway colonization with *P. aeruginosa*, and the capability of providing written informed consent. Pregnant and/or breastfeeding individuals were excluded per protocol, as were individuals with a diagnosis of cystic fibrosis, known acquired or hereditary immunodeficiency (such as HIV-positive patients), and staff members of the centers/institutes participating in the study.

The following patient characteristics were extracted from the patient records: sex, age, lung function, routine microbiologic analyses of respiratory samples prior to and up to 12 months after enrollment, records of previous antibiotic eradication attempts and antibiotic usage within three months prior to enrollment. However, a standardized classification into chronic versus intermittent *Pseudomonas* colonization was not feasible due to the variability of sampling frequency and availability of microbiologic analyses from clinical routine data.

**Fig. 1:**
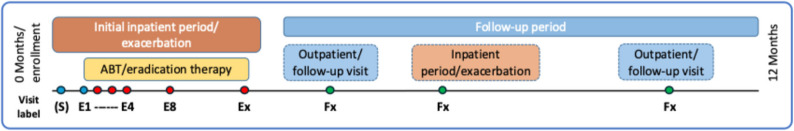
Graphical outline of the observational study design. Participants receiving inpatient treatment for COPD and/or NCFBE exacerbation and with known or newly detected *P. aeruginosa* colonization were enrolled. Lower respiratory tract samples (sputum and bronchial aspirate, if bronchoscopy was performed) were collected prior to the initiation of antibiotic eradication therapy (corresponding visit label S if sample was collected at a screening visit > 24 h before initiation of therapy or E1 if collected at day 0/directly prior to the initiation of antibiotic therapy), followed by high-frequency longitudinal sampling after 1, 2, 3 and 7 days of therapy (corresponding visit labels E2-E4, E8) as well as at the last day/at the end of antibiotic therapy (corresponding visit label Ex, where x is the day of therapy following the prior naming logic). Within a follow-up period of 12 months, any subsequent outpatient follow-up visits or inpatient periods were used for the collection of follow-up samples (corresponding visit labels Fx, where x is the month after enrollment)

Lower respiratory tract samples (sputum and bronchial aspirates, if available/bronchoscopy was performed) were collected from study participants during the initial inpatient treatment period. In case an antibiotic eradication therapy was initiated, sputum samples were collected at a high frequency during the treatment period (day 0/pre-treatment, days 1, 2, 3, 7, and at the end of antibiotic therapy). If participants could not produce a sufficient sample or per-protocol processing was not possible on a given date, sample collection on other days in the course of therapy was allowed in a pragmatic approach to ensure the availability of longitudinal samples. Participants were followed for a time frame of 12 months. Any outpatient follow-up visits or further inpatient treatment periods within this time frame were used for the collection of follow-up respiratory samples (Fig. 1).

Since this study was designed to be observational, enrollment did not exert any influence on the medical care and treatment (decision for or against ABT/eradication therapy, choice of antibiotic(s) or duration of therapy, time point of initiation of antibiotic therapy, medical follow-up with specialists, etc.).

### Sample processing

The collected lower respiratory samples were incubated (ratio 1:2) with dithiothreitol (Sputolysin, Merck KGaA, Germany) for 15 min at room temperature on a roller-mixer. After aliquoting, samples were stored at -80 °C until transport to the microbiological lab at the University of Luebeck for further analyses. Upon arrival, one sterile inoculation loop was used to streak the sample on MacConkey agar (bioMérieux S.A., France) for the selective isolation of Gram-negative bacteria and Cetrimide agar (Fisher Scientific GmbH, Germany) for the selective isolation of *P. aeruginosa*. Plates were incubated at 37 °C for up to 72 h. Additionally, 200 µl of the sample was inoculated in liquid enrichment thioglycollate broth (BD, United States), which was incubated at 37 °C for 24–72 h. When visual growth in liquid media was observed, positive broths were inoculated in MacConkey agar and incubated at 37 °C for up to 24 h. The Matrix-Assisted Laser Desorption Ionization-Time of Flight Mass Spectrometry (MALDI-TOF MS) was utilized to identify up to 10 *P. aeruginosa* colonies per sample, which were preserved in Tryptic Soy Broth with 15% glycerol and stored at − 80 °C.

### Resistance phenotyping

All isolates were cultivated on 5% sheep blood-enriched Columbia agar plates (bioMérieux S.A., France) overnight at 37 °C from glycerol stocks. Phenotypic susceptibility testing was conducted by broth microdilution following the manufacturer’s instructions for the MICRONAUT-S Pseudomonas MIC panel from the MICRONAUT system (Bruker Corporation, United States). Seventeen antimicrobial substances and/or combinations (in mg/L: meropenem (0.125-16), imipenem (1–8), ciprofloxacin (0.0625-8), levofloxacin (0.125-8), cefepime (1–8), ceftazidime (0.25-32), ceftazidime/avibactam (1/4–8/4), ceftolozane/tazobactam (1/4–8/4), amikacin (4–32), gentamicin (0.25-32), tobramycin (0.25-32), aztreonam (1–16), piperacillin (4–32), piperacillin/tazobactam (1/4-128/4), trimethoprim/sulfamethoxazole (1/19 − 8/152), fosfomycin (16–128), and colistin (1–8)) were included in this layout. *Escherichia coli* ATCC 25922 and colistin-resistant *E. coli* NCTC 13846 were used as quality control strains. Antibiotic susceptibility was interpreted according to the EUCAST clinical breakpoints v 15.0.

### DNA extraction, sequencing, and bioinformatic analysis

Genomic DNA was extracted from an overnight culture on Columbia blood agar using the DNeasy Blood and Tissue minikit (Qiagen, Germany) following the manufacturer’s instructions. Library preparations were performed using the DNA Prep Kit (Illumina, United States) and sequenced using the NextSeq 2000 instrument (2 × 100 cycles).

Raw fastq files were trimmed for adapters and low-quality reads using fastp (v0·23·1 with parameters -q = 30 and -l = 45) [11] and assembled with SPAdes 3.15.5 (with the option —careful and—only-assembler) [12]. A curation of the draft genomes was performed by removing contigs with a length < 500 bp and/or coverage < 10×, and the quality of the assembly was assessed using Quast (v5·0·2) [13]. The species identification of each draft genome was done using mash (sub-command screen) by screening each draft genome against a database composed of a representative genome of each species present in the Microbial Genomes resource (https://www.ncbi.nlm.nih.gov/genome/microbes/). The complete draft genomes were processed through available databases using Abricate (https://github.com/tseemann/abricate) to identify antimicrobial resistance (NCBI, CARD, ARG-ANNOT, ResFinder, MEGARES databases) and AMRfinderplus for point mutations [14–16]. Genomes were annotated using Prokka v.1.14.5 [17]. In order to compare the isolates patient-wise, one isolate of the first timepoint with the best N50 and completeness was used as reference for the single-nucleotide polymorphisms (SNPs) calling using snippy and gubbins to obtain a phylogenetic tree. The definition of clonal lineages was based on core-genome SNP distances, with isolates considered to belong to the same clonal lineage in one patient if they differed by ≤ 5000 SNPs, as described by Eklöf et al. (2022) [10].

## Results

### Population and clinical course of *P. aeruginosa* infection

A total of 30 patients met the inclusion criteria of this study and were enrolled between November 2020 and October 2023. Of these, 25 participants received in-hospital antibiotic eradication therapy. Longitudinal sampling of respiratory samples was successful in 22 patients, who therefore constituted the analysis cohort (Table 1). The median age was 69 years and 14 participants were male. All patients had chronic respiratory conditions (NCFBE, COPD, or both) qualifying for study inclusion. The median time between the first documentation of *P. aeruginosa* isolation from respiratory samples and the initiation of ABT/eradication therapy was one month. Available microbiological records suggested heterogeneous patterns of prior *P. aeruginosa* detection across the cohort, with some patients showing evidence of repeated prior detection, while others had a first documented detection shortly before study inclusion (see Supplementary dataset). Altogether, 91 respiratory samples were obtained before, during, and after eradication therapy.

**Table 1:** Baseline demographic and clinical characteristics of the analysis cohort

Participants enrolled, *n*	30
Received in-hospital eradication therapy, n	25
Analysis cohort, n	22
Age, years	Median: 69 (range 28–88)
Sex (M/F), n	14/8
Respiratory condition, n (%)	NCFBE: 11 (50%) COPD: 5 (22.7%) COPD + NCFBE: 6 (27.3%)
Time from first PA isolation to ABT, months	Median: 1 (range: < 1 months – > 3 years)
Duration of PA colonization > 3 months, n (%)	6 (27.3%)
Previous eradication attempts*, n (%)	6 (27.3%)
Any antibiotic therapy ≤ 3 months prior, n (%)	9 (40.9%)
Respiratory samples collected, n	Before ABT: 13 During ABT: 66 Follow-up samples: 12 Total: 91

*ABT* antibiotic therapy, *COPD* chronic obstructive pulmonary disease, *NCFBE* non-cystic fibrosis bronchiectasis, *PA* *Pseudomonas aeruginosa*

*Defined as ≥ 7 days of anti-pseudomonal antibiotic therapy

**Fig. 2:**
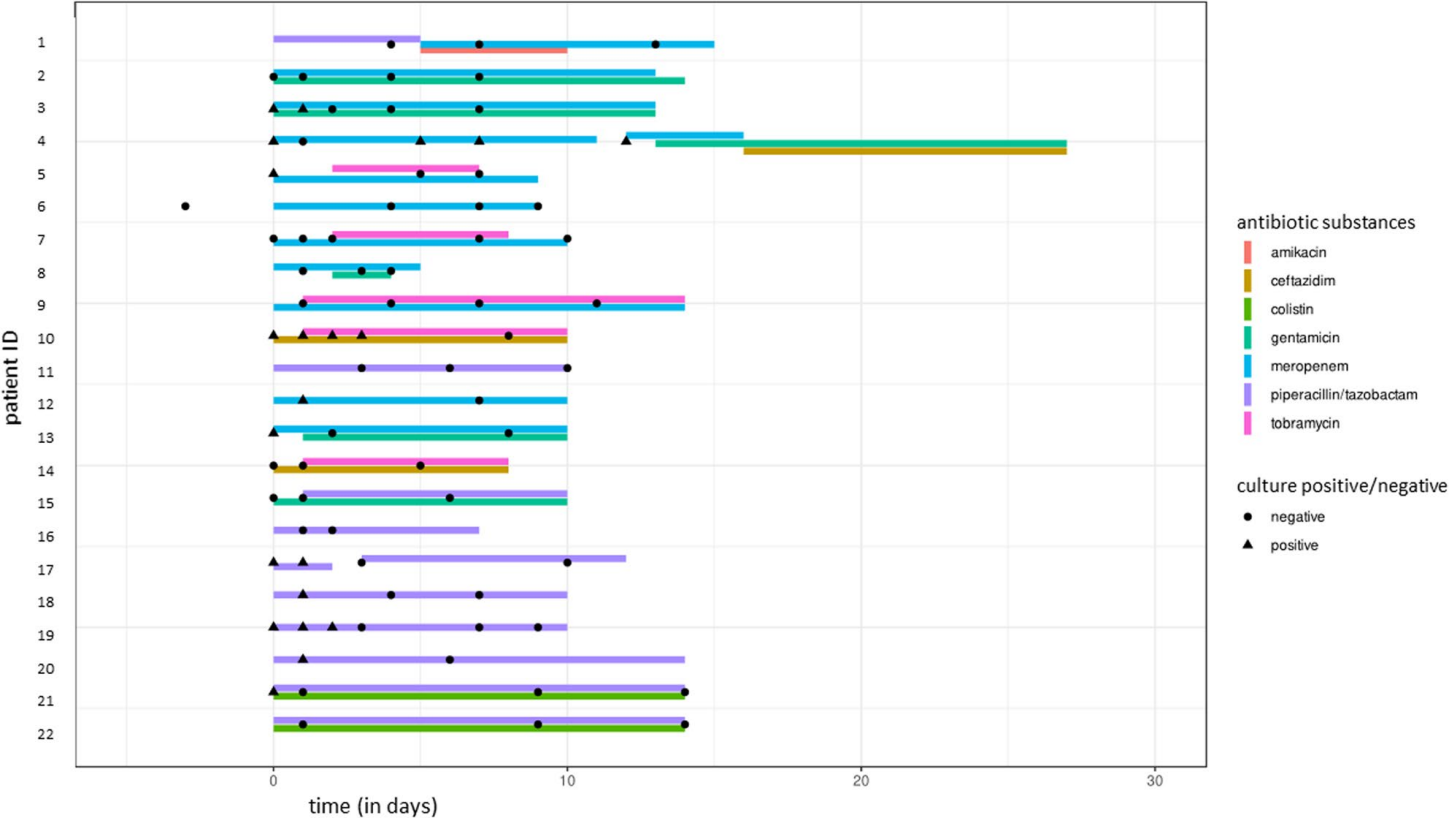
Overview of sample collection prior to and/or during the administration of eradication therapy for diagnosed *P. aeruginosa* colonization in the hospital. The majority of patients received either meropenem (often in combination with gentamicin or tobramycin) or piperacillin/tazobactam. Amendments to the antimicrobial dosage during the therapeutic regimen are indicated by a gap (patients 4 and 17). Of the 79 samples obtained from 22 patients, 26.6% (*n* = 21) from 11 patients were culture-positive for *P. aeruginosa*

*P. aeruginosa*-targeting ABT was administered based on routine microbiological analysis results/antimicrobial susceptibility tests and was at the sole discretion of the treating physician. The doses of the administered antibiotics are fully displayed in the data supplement (Supplementary dataset, sheet S1). The median duration of ABT eradication therapy was 10 days (minimum 5, maximum 27 days). All patients received an intravenous (i.v.) beta-lactam component, most frequently meropenem (*n* = 10/22, 45.5%) or piperacillin/tazobactam (*n* = 10/22, 45.5%) at initiation of therapy (Fig. 2). Combination therapy was initiated at the start of treatment in 8 (36.4%) patients, defined as an intravenous beta-lactam plus an inhaled or intravenous aminoglycoside, or inhaled colistin. In 5 (22.7%) cases, an intravenous or inhaled aminoglycoside was added to the beta-lactam later during therapy. In one case (4.5%), the beta-lactam was escalated from piperacillin/tazobactam to meropenem (Fig. 2).

Colistin was applied via inhalation (p.i.) only (*n* = 2, 9.1%), while the aminoglycosides gentamicin (*n* = 2, 9.1% i.v., *n* = 3, 13.6% p.i.) and tobramycin (*n* = 3, 13.6% i.v., *n* = 2, 9.1% p.i.) were applied both intravenously and via inhalation. Amikacin (*n* = 1, 4.5%) was applied intravenously only.

Despite all patients being enrolled based on proven airway colonization by *P. aeruginosa*, 11/22 (50%) patients were found to be culture-negative in the samples collected within the time frame of ABT/eradication therapy. Of the 11 patients who tested positive for *P. aeruginosa*, 6 were found to be positive on a single occasion only, either before or in the first 24 h of ABT. A total of 4 patients yielded 3 or more *P. aeruginosa*-positive culture results over time. In 3 of them (patients 4, 10, and 19), at least three consecutive samples were culture-positive. Patient no. 3, in contrast, tested positive in 2 consecutive sputum samples collected during the initial hospitalization period and again in a follow-up sample obtained 221 days after the first positive result. Among the 11 patients with *P. aeruginosa*-positive cultures at baseline or during the early course of therapy, short-term microbiology eradication, defined as a *P*. *aeruginosa*-negative culture in the last sample obtained during or at the end of antibiotic treatment, was achieved in 10 of 11 patients (90.9%). Across the entire study cohort, *P. aeruginosa* cultures were negative during or at the end of antibiotic treatment in 21 of 22 patients (95.5%), with the exception of patient no. 4. This patient received a prolonged course of antibiotics due to an initial treatment failure caused by inadvertent underdosing of meropenem. In this participant, sputum samples could only be collected during the course of meropenem therapy; no further suitable samples could be produced during a subsequent treatment period with gentamicin and ceftazidime.

For the purpose of this study, we further defined mid-term eradication success as continued *Pseudomonas*-negativity in all available follow-up respiratory microbiological analyses from this study or clinical routine within 12 months of study enrollment. For 8/22 (36.7%) patients, no suitable follow-up data were available. Of those with available follow-up data, 8/14 (57.1%) stayed *Pseudomonas*-negative, whilst 6/14 (42.9%) were tested *Pseudomonas*-positive again within 12 months of follow-up. Of the 8 participants with mid-term eradication success, 7 (87.5%) had received a combination of 2 antibiotics for at least part of their ABT cycle, whereas the majority of those with mid-term eradication failure had received monotherapy (*n* = 4/6, 66.6%). Further detailed information regarding patient and treatment characteristics and individual samples is provided in the Supplementary dataset (sheet S1).

### Antimicrobial susceptibility testing

In order to investigate the antimicrobial resistance of *P. aeruginosa* isolates collected before and during ABT, antimicrobial susceptibility testing to a panel of antibiotics was conducted by measuring the minimum inhibitory concentration (MIC). The MIC values of 170 isolates from 11 patients for 17 antibiotic substances were determined in total.

**Fig. 3:**
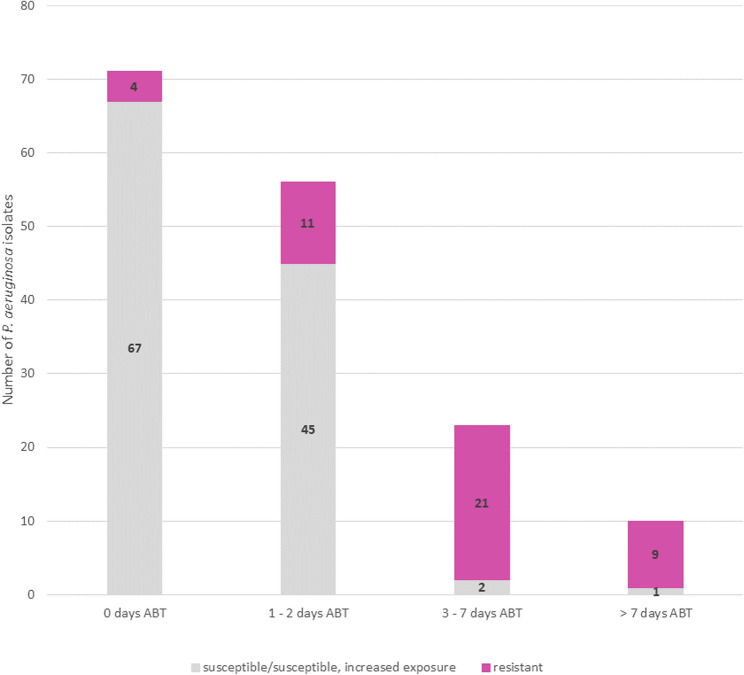
Overview of the number of *P. aeruginosa* isolates obtained from 11 patients grouped by time of antibiotic therapy (ABT). Isolates demonstrating resistance to a minimum of one antibiotic substance evaluated via broth microdilution are indicated in violet. While only 5.6% of strains isolated from samples taken before the administration of ABT exhibited resistance to at least one antibiotic substance, a higher proportion of isolates demonstrated resistance during the first 2 days, 3–7 days, and over 7 days of ABT (19.6%, 91.3%, and 90%, respectively)

The majority of *Pseudomonas* isolates were obtained from samples taken either before (*n* = 71/170, 41.8%) or in the first 2 days of eradication therapy (*n* = 56/170, 32.9%). While a small percentage of strains present before the initiation of ABT exhibited resistance to at least one antibiotic (5.6%), this proportion was higher in isolates obtained during treatment, with 19.6% showing resistance within the first 2 days, 91.3% between days 3 and 7, and 90% beyond 7 days of ABT (Fig. 3).

For 3 patients (5, 18, and 20), only a single isolate was obtained from the initial sample, preventing assessment of intra-host antimicrobial resistance heterogeneity. Among the remaining patients, intra-sample MIC variability was observed across multiple baseline isolate sets (first positive samples), including for fluoroquinolones and carbapenems. However, these MIC differences did not consistently result in categorical differences in EUCAST susceptibility interpretation (susceptible, susceptible with increased exposure, or resistant). In line with this, isolates from patients 4, 12, 17, 19, and 21 were susceptible or susceptible with increased exposure to all tested antimicrobial agents (Supplementary dataset, sheet S2). Phenotypic resistance variation in *Pseudomonas* prior to the initiation of antibiotic treatment was observed in only 3 patients. Patient 3 had 2 isolates resistant to imipenem (MIC > 4 mg/L) and colistin (MIC > 4 mg/L), respectively, while patients 10 and 13 each carried a ciprofloxacin-resistant isolate (MIC > 0.5 mg/L).

In relation to the isolation of *P. aeruginosa* from samples obtained prior to and during eradication therapy, only 3 patients were culture-positive for 2 or more days under ABT. Patient 4 received an inadvertent underdosing of meropenem for the first 11 days of ABT and was culture-positive for the longest period recorded, with positive cultures before (day 0) and during ABT (days 5, 7, and 12). While the ten isolates on day 0 were not resistant to any of the tested antibiotics, almost all isolates on day 5 (*n* = 10/10), day 7 (*n* = 10/10), and day 12 (*n* = 9/10) were resistant to imipenem. With regard to MIC values of meropenem over time, all isolates collected on day 0 had MICs of ≤ 0.125 mg/L. By day 5, a higher proportion of isolates exhibited meropenem MIC values of 2 mg/L (6 out of 10 isolates), and by day 7, 7 out of 10 isolates showed MICs of 2 mg/L, with 1 additional isolate exhibiting a MIC of 4 mg/L. Isolates obtained from the sample collected on day 12 of ABT exhibited lower meropenem MIC values (ranging from ≤ 0.125 mg/L to 0.5 mg/L), compared to isolates from the previous 2 sampling time points (Supplementary dataset, sheet S2). Two sputum samples obtained 231 and 236 days following the initial sampling during a subsequent hospitalization were found to be negative for *P. aeruginosa.*

Patient 10 received ceftazidime in combination with tobramycin for 10 days and remained culture-positive during the first 3 days of ABT. While only 1 ciprofloxacin-resistant isolate (10%) was found prior to the initiation of ABT, 2 (20%), 6 (60%), and 1 (33.3%) ciprofloxacin-resistant isolates were obtained from samples taken on days 1, 2, and 3 under ABT, respectively. In addition to the high ciprofloxacin resistance rate of 60% on day 2, 2 ciprofloxacin-resistant isolates were also resistant to levofloxacin (MIC > 2 mg/L) and levofloxacin plus imipenem, and 1 isolate was resistant to imipenem alone. Furthermore, 2 strains isolated from the sample taken after 24 h ABT were colistin-resistant. No *P. aeruginosa* isolates could be cultured from the sample taken on day 8 of ABT.

Patient 19 exhibited positive results for *P. aeruginosa* in the first 2 days of antibiotic treatment, with all isolates demonstrating susceptibility or susceptibility with increased exposure to tested antibiotics. Subsequent cultures (on days 7 and 9) from this patient were *Pseudomonas*-negative.

### Genomic heterogeneity of *P. aeruginosa*

**Fig. 4:**
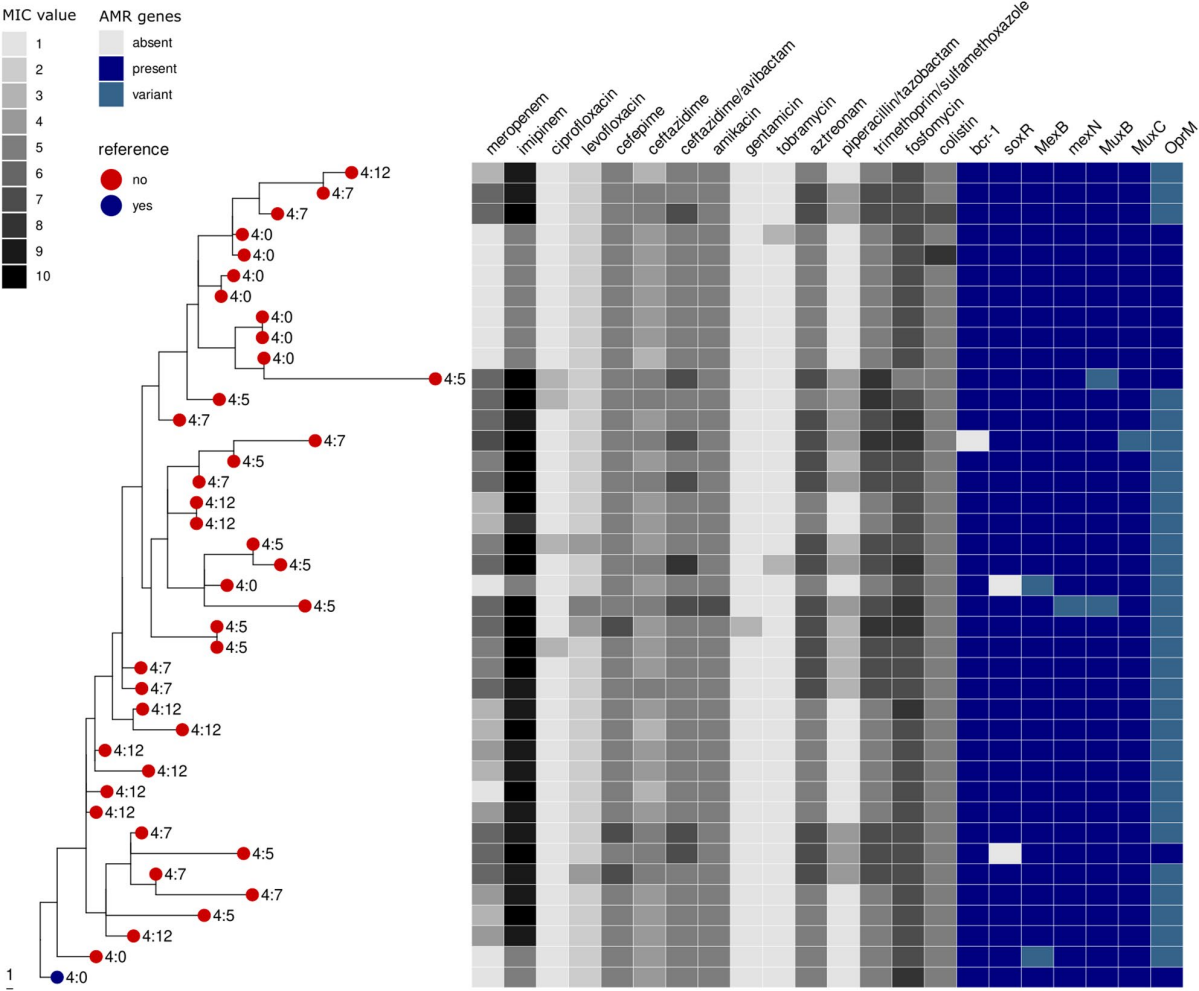
Phylogeny of 40 *P. aeruginosa* isolates obtained from patient 4, who remained culture-positive for the longest duration recorded in this study and received a subtherapeutic dose of meropenem during the first 11 days of eradication therapy. Minimum inhibitory concentration (MIC) values for each antibiotic are color-coded to visualize variation among isolates. The presence or absence of genes linked to influencing antibiotic resistance in *Pseudomonas* is shown for each isolate

In order to characterise the heterogeneity of *P. aeruginosa* before and during ABT within patients, the genomes of 117 isolates were sequenced (Supplementary dataset, sheets S3-S6). These isolates were collected from 4 patients (3, 4, 10, and 19) who were positive for *P. aeruginosa* in at least 3 lower respiratory tract samples (including follow-up sampling). A genetic analysis of the isolates revealed that all 4 patients were found to be colonized by a single *P. aeruginosa* lineage, with up to 237 SNPs differences between isolates from patient 3, 103 SNPs in patient 4, 106 SNPs in patient 10, and 18 SNPs in patient 19. *Pseudomonas* strains did not consistently segregate by sampling time within individual patients, suggesting that genomic heterogeneity was already present from the first sample and remained throughout antibiotic treatment (Fig. 4, Supplementary Material; Fig S1 to S3).

MIC values for individual antibiotics demonstrated variability both among isolates within single samples and longitudinally throughout treatment (Supplementary Material; Table S1). Overall, concordance between phenotypic antibiotic resistance and detected genotypic resistance determinants was limited. The majority of resistance genes identified were present across isolates from individual patients, irrespective of the sampling timepoint. In patient 4, variants of the outer membrane protein OprM (89.71% − 92.52% identity; primarily Val425Gly) were identified in conjunction with mexB genes (100% identity) in the majority of imipenem-resistant isolates (*n* = 27/29). Isolates from patients 3, 10, and 19 also carried OprM variants in combination with mexB genes (including an OprM Leu198fs mutation in patient 3, while no mutations were detected by snippy in patients 10 and 19). However, the majority of these isolates did not exhibit phenotypic imipenem resistance. Compared to the original susceptible isolates used as reference genomes for each of the 4 patients, SNPs were identified in the remaining isolates. With the exception of imipenem resistance in patient 4, no consistent associations between mutations detected by snippy and elevated MIC values were observed in the patient isolates (Supplementary dataset, sheets S7-S10).

## Discussion

Here, we present data about the intra-patient heterogeneity of *P. aeruginosa* and its resistance dynamics in response to eradication therapy in a real-world observational cohort of colonized COPD and NCFBE patients. To our knowledge, this is the first study to combine detailed sampling during antibiotic therapy with extensive isolate sampling, comprehensive phenotypic resistance profiling, and genomic sequencing of isolates, allowing for a detailed exploration of intra-patient *Pseudomonas* dynamics.

We observed very low resistance rates in pre-eradication *P. aeruginosa* isolates, with 94.4% of isolates (*n* = 67/71) exhibiting no phenotypic resistance against the panel of antibiotics tested. This finding is overall consistent with two previous studies indicating that the majority of *P. aeruginosa* isolates from patients with COPD (*n* = 65/112, 58%) [18] and NCFBE (*n* = 67/95, 70.5%) [6] showed no resistance to tested antibiotics. However, in contrast to our work, the isolates were not exclusively collected prior to the initiation of antibiotic/eradication treatment in these studies, possibly accounting for the moderately higher resistance rates reported.

To explore the intra-host heterogeneity of *Pseudomonas* colonization, we collected up to 10 randomly chosen isolates from patient samples containing *Pseudomonas* in this study. Van den Bossche et al. previously reported a high intra-sample MIC heterogeneity of *P. aeruginosa* from patients with cystic fibrosis, stating that the pooling of nine isolates obtained from a single sample led to a decrease of the intra-sample heterogeneity and the generation of more consistent antimicrobial susceptibility testing results [19]. While we also observed MIC heterogeneity among isolates obtained from single samples, this was based on a limited number of samples (18 samples from 8 patients) and should therefore be interpreted with caution. Within this context, intra-host phenotypic resistance heterogeneity according to EUCAST susceptibility categories appeared limited in our cohort of patients with COPD and/or NCFBE. The high rate of short-term eradication among patients with *P. aeruginosa*-positive cultures detected at baseline or during ABT (10/11 patients, 90.9%) supports the general effectiveness of the antibiotic treatment regimens currently applied in clinical routine, even in the presence of some level of resistance heterogeneity in some patients. Though our data is indicative of a potentially higher mid-term eradication success of antibiotic combination therapy compared to monotherapy, the number of analyzable cases in our study remains too low for a valid statistical inference.

We observed one participant who received an inadvertent underdosing of meropenem, resulting in the recurrent isolation of imipenem-resistant isolates and a prolonged course of ABT. This highlights the importance of the pharmacokinetics/pharmacodynamics relationship and sufficient antibiotic dosing in the context of treatment of the highly adaptive pathogen *P. aeruginosa*. Recurrent detection of *P. aeruginosa* in patients with COPD has previously been associated with the persistence of the same clonal lineage for up to one year [10]. Another study reported on *Pseudomonas* colonization by multiple strains in ICU patients (*n* = 12/35) and demonstrated that resistance evolved rapidly in these patients through selection of pre-existing strains [9]. In the present study, all four patients who produced three or more *Pseudomonas*-positive cultures during the ABT period were found to be colonized by a single clonal lineage. The definition of clonal lineages in this study was based on a ≤ 5000 SNP threshold, as previously described in the literature [10]. While this cutoff allows for comparability with prior studies, it represents a relatively broad definition and may obscure finer-scale intra-host diversification. However, SNP distances observed within patients were lower (≤ 250 SNPs), indicating limited genomic divergence in our cohort. The overall low antimicrobial resistance rate observed in all *Pseudomonas*-positive samples in our cohort, as well as the high rate of short-term eradication success, may suggest that the remaining half of our COPD/NCFBE cohort, which could not produce *Pseudomonas*-positive samples during the course of antibiotic therapy, was also colonized by only one clonal lineage. Potential factors that may promote colonization by multiple *P. aeruginosa* lineages, such as high-pressure environments like ICUs or the presence of additional comorbidities, warrant further investigation beyond the scope of this study. In such settings, resistance dynamics and treatment outcomes may differ substantially, particularly in severe respiratory infections such as ventilator-associated pneumonia, where pathogen burden, prior antibiotic exposure, and the healthcare environment are likely to play a greater role. Recent literature has also highlighted the clinical relevance of newer anti-pseudomonal agents, including ceftolozane-tazobactam, ceftazidime-avibactam, and cefiderocol, in these high-risk contexts [20]. These observations underline that antimicrobial resistance dynamics are highly context-dependent and that our findings should not be directly generalized beyond the relatively low-resistance cohort studied here.

While genomic analysis identified SNPs that aligned with imipenem resistance, it was insufficient on its own to accurately predict phenotypic resistance across all antibiotics in our study. This finding highlights the limitations of relying solely on sequencing-based approaches for antimicrobial resistance profiling in *Pseudomonas*. Dolgusevs et al. reported on significant discrepancies when phenotypic data were compared to genotypic data for β-lactams, fluoroquinolones, and aminoglycosides [21]. Recent data suggest that environmental stressors, such as oxidative stress and nutrient limitation, can induce phenotypic resistance by transiently activating efflux systems and other adaptive responses [22, 23]. This contributes to resistance phenotypes that are not readily explained by genomic data alone. Together, these findings highlight that phenotypic resistance in *P. aeruginosa* is often governed by dynamic and context-dependent regulatory processes, which may not be fully captured by standard genomic approaches, thereby limiting the predictive value of genotype-based resistance profiling. Consequently, phenotypic testing remains essential for the accurate assessment of resistance profiles in *P. aeruginosa*, thereby ensuring that clinicians are guided by reliable data when selecting effective treatment options.

The present study also has limitations. Although confirmed *P. aeruginosa* colonization was an inclusion criterion, only 50% of enrolled COPD/NCFBE patients were able to produce samples that were culture-positive for *P. aeruginosa* within the time frame of ABT. Compared with studies in cystic fibrosis (CF), this may reflect differences in airway disease pathophysiology and colonization dynamics between COPD/NCFBE and CF, potentially including lower bacterial density, lower expectorated sputum volume or a more transient presence of *P. aeruginosa* in COPD/NCFBE (intermittent colonization). However, in all patients, the initiation of eradication therapy by the treating physician was based on a suspected chronic colonization/infection. Notably, participation in this observational study did not exert any influence on the choice for or against ABT/eradication therapy in the patients, nor were the choice of antibiotic(s), the timepoint of initiation, or the duration of therapy influenced by study enrollment. The respiratory samples consisted mainly of sputum samples; bronchial aspirates were only collected if bronchoscopy was performed as a measure of routine clinical care, irrespective of study participation. This ensured a minimal burden for the participating patients and a low invasiveness associated with material collection. Consequently, it can be deduced that the sampling of biomaterials (e.g. sputum) in this study was subject to the same pre-analytical challenges observed in routine microbiological diagnostics for pulmonary patients. Therefore, a potential sampling error (low-quality/amount sputum samples being provided before the start of ABT due to time constraints that come with high-frequency sampling and treatment workflows of routinely admitted patients) cannot be excluded in individuals in which no *P. aeruginosa*-positive sample could be collected within the time frame of ABT, despite the utmost care being exercised in the collection, transportation and processing of samples. This limitation may have contributed to an overestimation of the observed short-term eradication rates and should therefore be considered when interpreting treatment success in this cohort. In this study, the number of enrolled patients was limited because recruitment was restricted to two clinical centers within the same region. Furthermore, this study was initiated and conducted during the COVID-19 pandemic during which the number of COPD exacerbations and inpatient treatments for COPD decreased substantially [24], complicating recruitment efforts, and COVID-19-related restrictions on outpatient care at the participating clinical centers hindered the scheduling of follow-up outpatient visits and collection of follow-up sputum samples. COPD and NCFBE patient cohorts exhibit a significant overlap [25], and therefore we decided to include both disease cohorts in this study. However, the study is underpowered to delineate clinically meaningful differences in *P. aeruginosa* colonization between COPD-only and NCFBE-only patients or between antibiotic regimens.

Despite these limitations, our data suggest that phenotypic resistance heterogeneity was observed within individual samples, while the overall frequency of antimicrobial resistance and the extent of intra-host phenotypic resistance heterogeneity appeared relatively limited in this cohort of COPD/NCFBE patients with recently detected *Pseudomonas* colonization. Antibiotic treatment regimes currently applied in clinical practice for eradication therapy exhibit a high short-term effectiveness, as demonstrated by rapid culture negativity in the course of therapy, as observed in our cohort. By whole-genome sequencing of longitudinally collected isolates, we demonstrated that COPD/NCFBE patients are likely foremost colonized by a single clonal lineage of *P. aeruginosa*. Both our phenotypic resistance profiling as well as genome sequencing revealed adaptive resistance dynamics under antibiotic pressure, whilst this did not diminish short-term treatment efficacy in our cohort. However, in the case of antimicrobial underdosing, resistant isolates were observed, which may reflect either selection of pre-existing resistant subpopulations or adaptive resistance evolution, highlighting the adaptive capabilities of *Pseudomonas aeruginosa* and the necessity to carefully select appropriate antimicrobial treatment regimens. It remains uncertain whether mid-term eradication failures (re-detection of *P. aeruginosa* within 12 months after eradication therapy) result from bacterial persistence in the respiratory tract or from re-infection with new strains acquired from external sources. In our study, one patient tested positive again for *P. aeruginosa* more than 200 days after eradication therapy, with the isolates belonging to the same clonal lineage, suggesting bacterial persistence rather than re-infection. However, we cannot exclude re-exposure from a persistent environmental source, such as contaminated sinks or drains, which have been described as long-term reservoirs for *P. aeruginosa*, although such transmission events appear to be infrequent [26]. Genotyping may currently offer only limited additional value in routine microbiological diagnostics of COPD/NCFBE patients, as the overall correlation between detected resistance determinants and phenotypic susceptibility was low in our cohort. This finding should be interpreted with caution, because most isolates were susceptible and resistance in *P. aeruginosa*, including mechanisms involving efflux pumps, can complicate genotype-phenotype predictions. The high rate of treatment success observed in our cohort suggests that antimicrobial susceptibility testing of a single isolate could be sufficient to inform antibiotic eradication therapy in COPD/NCFBE patients, in contrast to the more diverse *P. aeruginosa* populations found in CF patients [27, 28], and the resulting treatment recommendations [19]. These findings should be considered hypothesis-generating, given the limited sample size and the low baseline resistance observed in our cohort. Severe phenotypes, a history of treatment failure, or high-pressure environments such as the ICU that are not represented by our cohort, may necessitate more comprehensive approaches.

Further research in larger-scale cohorts is needed to elucidate the long-term dynamics of *P. aeruginosa* colonization in COPD/NCFBE, to assess the sustained impact of eradication therapy, and to identify optimal antimicrobial treatment strategies.

## Supplementary Information

Supplementary Material 1.

Supplementary Material 2.

Supplementary Material 3.

## Data Availability

The draft genomes presented in this study can be found in the NCBI Genbank repositories under the Bioproject PRJNA1419033.

## Abbreviations

ABT: Antibiotic therapy
CF: Cystic fibrosis
COPD: Chronic obstructive pulmonary disease
DRKS: German Clinical Trials Register
EUCAST: European Committee on Antimicrobial Susceptibility Testing
HIV: Human immunodeficiency virus
ICU: Intensive care unit
i.v.: Intravenous
MALDI-TOF MS: Matrix-assisted laser desorption ionization time-of-flight mass spectrometry
MIC: Minimum inhibitory concentration
NCFBE: Non-cystic fibrosis bronchiectasis
p.i.: Per inhalation
SNP: Single-nucleotide polymorphism
