# Supplementary material for: Antibiotic pressure does not uncover intra-host heterogeneity of Pseudomonas aeruginosa in patients with chronic lung disease
Source: BMC Microbiol. 2026 May 21;26:480. doi: 10.1186/s12866-026-05147-9 (PMC13192145; doi:10.1186/s12866-026-05147-9)

**Supplementary Material**

**Antibiotic pressure does not uncover intra-host heterogeneity of *Pseudomonas aeruginosa* in patients with chronic lung disease**

**Table of contents**

[**Supplementary dataset (separate Excel file).** Overview of patient cohort, antimicrobial susceptibility testing, and sequencing data analysis 2](#_Toc221183631)

[**Supplemental Table 1.** Overview of all clinical samples from which more than one *P. aeruginosa* isolate was recovered. The table presents the mean minimum inhibitory concentration (MIC) values and MIC ranges per sample for selected antibiotics. 3](#_Toc221183632)

[**Supplementary figure 1.** Phylogeny of 21 *P. aeruginosa* isolates obtained from patient 3 across three distinct sputum samples. 5](#_Toc221183633)

[**Supplementary figure 2.** Phylogeny of 33 *P. aeruginosa* isolates obtained from patient 10 across four distinct sputum samples 6](#_Toc221183634)

[**Supplementary figure 3.** Phylogeny of 23 *P. aeruginosa* isolates obtained from patient 19 across three distinct sputum samples. 7](#_Toc221183635)

**Supplementary dataset (separate Excel file).** Overview of patient cohort, antimicrobial susceptibility testing, and sequencing data analysis.

**Supplemental Table 1.** Overview of all clinical samples from which more than one *P. aeruginosa* isolate was recovered. The table presents the mean minimum inhibitory concentration (MIC) values and MIC ranges per sample for selected antibiotics. Antibiotic agents without available breakpoints (according to EUCAST clinical breakpoints v 15.0) for *P. aeruginosa* (gentamicin, fosfomycin, trimethoprim/sulfamethoxazole) and those demonstrating only 2-fold MIC variation across isolates (amikacin, tobramycin, ceftolozane/tazobactam) are not shown. For the purpose of mean MIC calculation, values reported as “≤X” were treated as equal to X, while values reported as “>8” were treated as 16. Fold-changes in MIC values between isolates from the same sample are visually represented using a color gradient: the lightest shade (rose) indicates a 2-fold change, while the darkest shade (red) corresponds to a 32-fold change. If at least one isolate from a sample exhibited resistance to a given antibiotic, the corresponding table cell was outlined in black to denote phenotypic resistance.

|  |  | Antibiotic substances, mean MIC in mg/L (MIC range) | | | | | | | | | | |
| --- | --- | --- | --- | --- | --- | --- | --- | --- | --- | --- | --- | --- |
| Sample IDs | n | MER | IMI | CIP | LEV | CEP | CAZ | CAA | AZT | PIP | PIP/TAZ | COL |
| Patient03_s_E1 | 10 | 0.2125  (≤0.125–0.25) | 2.2  (≤1–8) | 0.125 | 0.25 | 2.4  (≤1–4) | 3.6  (2–8) | 3/4  (2/4–4/4) | 8 | ≤4 | 7.2/4  (4/4–8/4) | 2.5  (≤1 – >8) |
| Patient03_s_F6 | 10 | 0.15  (≤0.125–0.25) | 1.8  (≤1–2) | 0.25625  (≤0.0625–1) | 0.5125  (≤0.125–2) | 1.3  (≤1–4) | 2.225  (≤0.25–8) | 1.1  (≤1/4–2/4) | 2.6  (≤1–8) | 5.2  (≤4 – >8) | 4.8/4  (≤1/4–32/4) | ≤1 |
| Patient04_s_E1 | 10 | ≤0.125 | ≤1 | ≤0.0625 | ≤0.125 | ≤1 | 0.95  (0.5–1) | ≤1/4 | ≤1 | ≤4 | ≤1/4 | 1.3  (≤1–4) |
| patient04_s_E6 | 10 | 1.525  (0.25–2) | >8 | 0.0875  (≤0.0625-0.125) | 0.1875  (≤0.125–0.5) | 1.1  (≤1–2) | 1.5  (1–2) | 1.6/4  (≤1/4–4/4) | 3.4  (≤1–4) | ≤4 | 2.9/4  (≤1/4–4/4) | ≤1 |
| patient04_s_E8 | 10 | 1.95  (0.5–4) | 11.2  (8 – >8) | ≤0.0625 | 0.1375  (≤0.125–0.25) | 1.2  (≤1–2) | 1.7  (1-2) | 1.4/4  (≤1/4–2/4) | 3.7  (≤1–4) | ≤4 | 3.3/4  (≤1/4–4/4) | 1.1  (≤1–2) |
| patient04_s_E13 | 10 | 0.3125  (≤0.125–0.5) | 10  (4 – >8) | ≤0.0625 | ≤0.125 | ≤1 | 0.85  (0.5–1) | ≤1/4 | ≤1 | ≤4 | ≤1/4 | ≤1 |
| Patient10_s_E1 | 10 | ≤0.125 | 1.5  (≤1–2) | 0.2125  (≤0.0625–1) | 0.4125  (≤0.125–2) | 1.1  (≤1–2) | 1.2  (1–2) | ≤1/4 | ≤1 | ≤4 | ≤1/4 | 1.3  (≤1–2) |
| Patient10_s_E2 | 10 | 0.1375  (≤0.125–0.25) | 2  (≤1–4) | 0.4875  (0.125–1) | 1.025  (≤0.125–2) | ≤1 | 1.15  (0.5–2) | 1.7/4  (≤1/4–8/4) | 2.4  (≤1–8) | ≤4 | ≤1/4 | 4.3  (≤1 – >8) |
| Patient10_s_E3 | 10 | 0.15  (≤0.125–0.25) | 3.6  (≤1 – >8) | 0.76875  (≤0.0625–2) | 1.625  (≤0.125–4) | 1.4  (≤1–4) | 1.3  (1–2) | ≤1/4 | 1.3  (≤1–4) | ≤4 | 1.2/4  (≤1/4–2/4) | 1.2  (≤1–2) |
| Patient10_s_E4 | 3 | ≤0.125 | 1.3  (≤1–2) | 0.67  (0.5–1) | 1.3 (1–2) | ≤1 | 1.3  (1–2) | ≤1/4 | ≤1 | ≤4 | ≤1/4 | ≤1 |
| Patient12_s_E2 | 10 | ≤0.125 | ≤1 | ≤0.0625 | ≤0.125 | ≤1 | 0.475  (≤0.25–0.5) | ≤1/4 | ≤1 | ≤4 | ≤1/4 | ≤1 |
| patient13_s_E1 | 10 | 0.15  (≤0.125–0.25) | 1.6  (≤1–2) | 0.225  (0.125–1) | 0.5375  (≤0.125–1) | 2 | 3.6  (2–4) | 2.1/4  (≤1/4–4/4) | 8 | ≤4 | 5.2/4  (4/4–8/4) | 1.1  (≤1–2) |
| patient17_s_E1 | 10 | 0.275  (≤0.125–0.5) | 2.8  (2–4) | ≤0.0625 | ≤0.125 | 1.1  (≤1–2) | 1.05  (0.5–2) | ≤1/4 | ≤1 | ≤4 | 1.7  (≤1/4–4/4) | ≤1 |
| patient17_s_E2 | 10 | 0.2375  (≤0.125–0.25) | 1.9  (≤1–2) | 0.1  (≤0.0625–0.125) | ≤0.125 | ≤1 | 1.125  (≤0.25–2) | ≤1/4 | 2.2  (≤1–4) | ≤4 | 2.3/4  (≤1/4–4/4) | ≤1 |
| patient19_s_E1 | 10 | ≤0.125 | 2.5  (≤1–4) | 0.10625  (≤0.0625–0.125) | 0.1375  (≤0.125–0.25) | ≤1 | 1.15  (0.5–2) | ≤1/4 | ≤1 | ≤4 | 1.1/4  (≤1/4–2/4) | 1.1  (≤1–2) |
| patient19_s_E2 | 10 | 0.1375  (≤0.125–0.25) | 1.3  (≤1–2) | 0.06875  (≤0.0625–0.125) | ≤0.125 | ≤1 | 1.25  (0.5–4) | ≤1/4 | 1.3  (≤1–4) | ≤4 | 1.1/4  (≤1/4–2/4) | ≤1 |
| patient19_s_E3 | 3 | ≤0.125 | 2  (≤1–4) | 0.17  (≤0.0625–0.125) | ≤0.125 | ≤1 | 1.3  (1–2) | 1.3/4  (≤1/4–2/4) | ≤1 | ≤4 | ≤1/4 | ≤1 |
| patient21_s_E1 | 10 | ≤0.125 | 2.2  (2–4) | ≤0.0625 | ≤0.125 | 1.2  (≤1–2) | 1.2  (1–2) | ≤1/4 | ≤1 | ≤4 | ≤1/4 | ≤1 |

Abbreviations: n = number of *P. aeruginosa* isolates; MER = meropenem; IMI = imipinem; CIP = ciprofloxacin; LEV = levofloxacin; CEP = cefepime; CAZ = ceftazidime; CAA = ceftazidime/avibactam; AZT = aztreonam; PIP = piperacillin; PIP/TAZ = piperacillin/tazobactam; COL = colistin.

**Supplementary figure 1.** Phylogeny of 21 *P. aeruginosa* isolates obtained from patient 3 across three distinct sputum samples. Minimum inhibitory concentration (MIC) values for each antibiotic are color-coded to visualize variation among isolates. The presence or absence of efflux pump genes MexB and OprM within the genomic content of each isolate is also indicated.


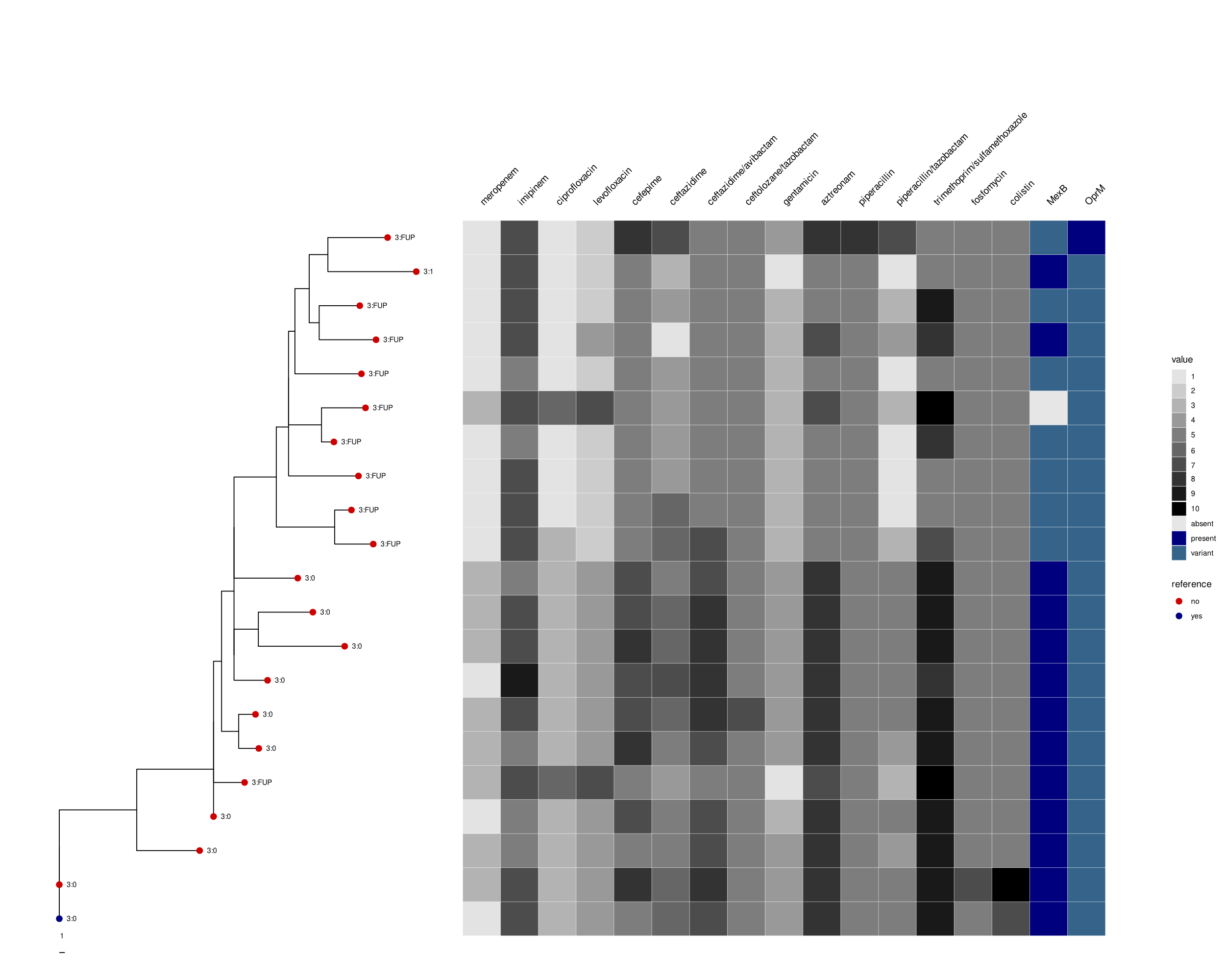


**Supplementary figure 2.** Phylogeny of 33 *P. aeruginosa* isolates obtained from patient 10 across four distinct sputum samples. Minimum inhibitory concentration (MIC) values for each antibiotic are color-coded to visualize variation among isolates. The presence or absence of genes linked to influencing antibiotic resistance in *Pseudomonas* is shown for each isolate.


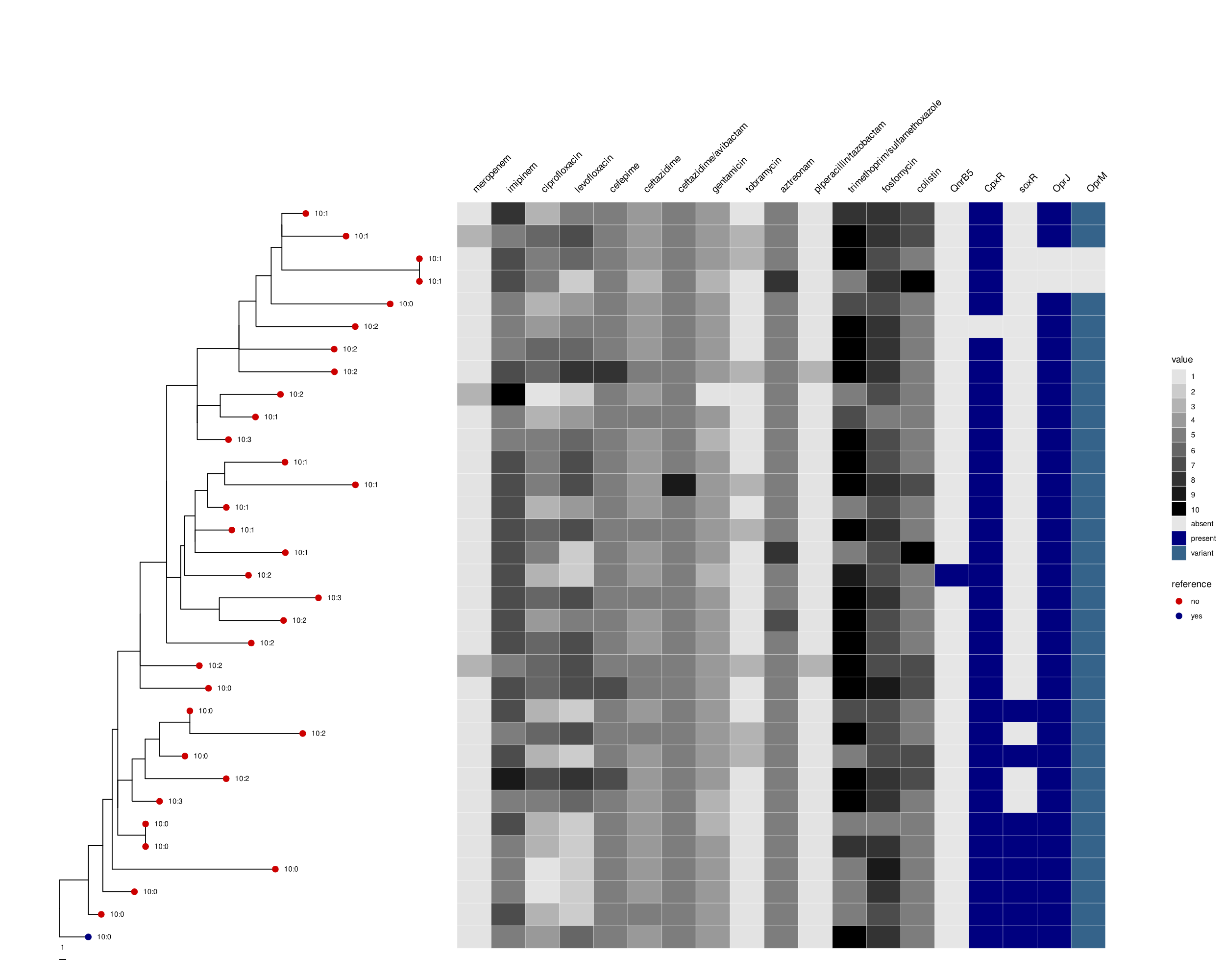


**Supplementary figure 3.** Phylogeny of 23 *P. aeruginosa* isolates obtained from patient 19 across three distinct sputum samples. Minimum inhibitory concentration (MIC) values for each antibiotic are color coded to visualize variation among isolates. The presence or absence of genes linked to influencing antibiotic resistance in *Pseudomonas* is shown for each isolate.


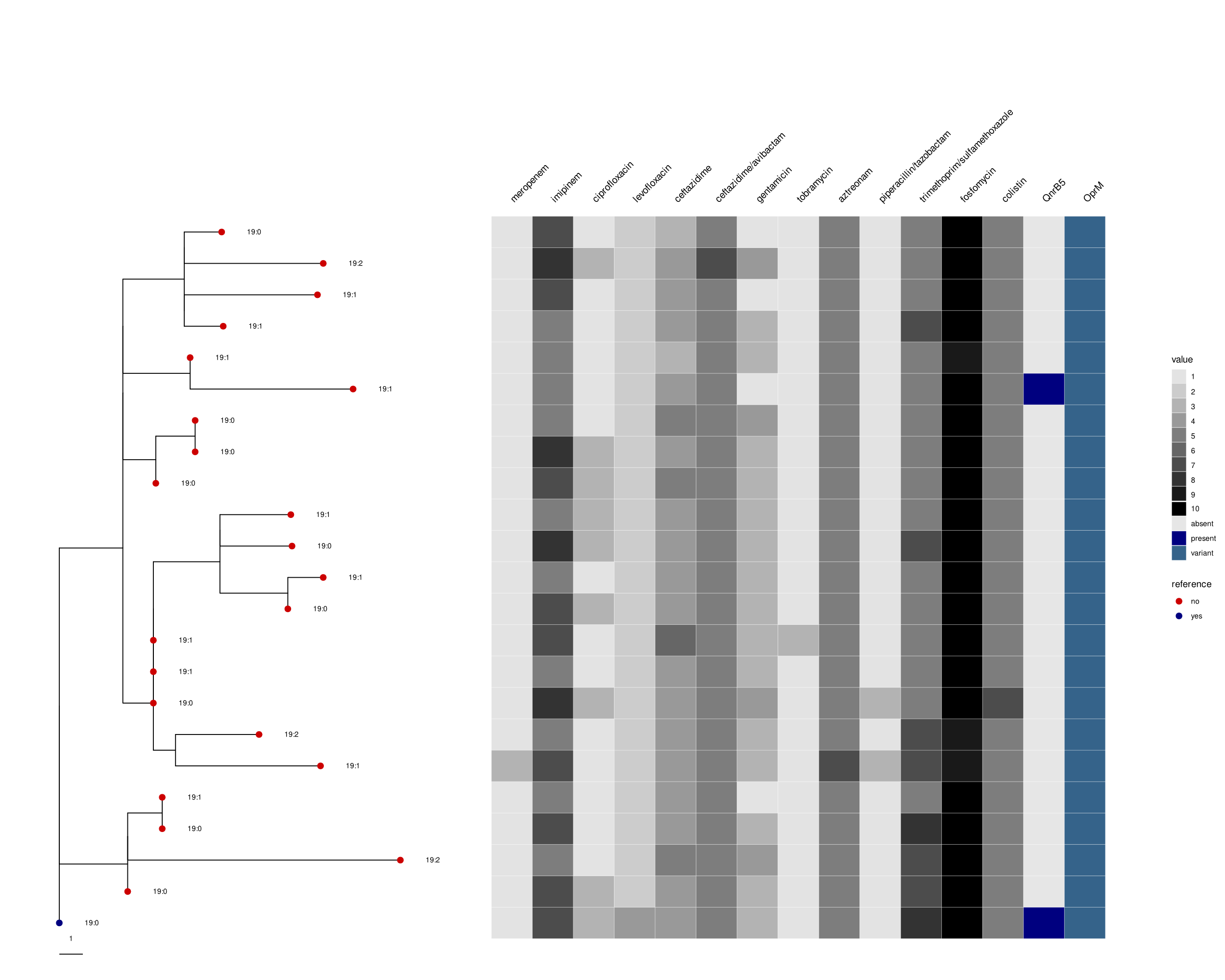

Supplement: Supplementary file 1 — Supplementary Material 1. [file 12866_2026_5147_MOESM1_ESM.docx]
